# The role of Wnt/β-catenin-lin28a/let-7 axis in embryo implantation competency and epithelial-mesenchymal transition (EMT)

**DOI:** 10.1186/s12964-020-00562-5

**Published:** 2020-07-11

**Authors:** Qian Li, Juanzi Shi, Weimin Liu

**Affiliations:** 1 Department of Obstetrics and Gynaecology, Laboratory Block, The University of Hong Kong, 21 Sassoon Road, Pokfulam, Hong Kong, SAR China; 2 Assisted Reproductive Center, Women & Children’s Hospital of Northwest China, 73 Hou zai Road, Xi’an, China

**Keywords:** Wnt/β-catenin signaling, Lin28a protein, Mir-let-7 family, Implantation competency, Embryonic EMT

## Abstract

**Background:**

The pre-implantation embryo in a competent status and post-implantation fully differentiation of the inner cell mass (ICM) and trophectoderm (TE) are prerequisites of successful implantation. Type I embryonic epithelial-mesenchymal transition (EMT) involves in these processes. A high level of the mir-let-7 family was found in the dormant mouse embryo of implantation failure in our previous study. Besides, its natural inhibitor lin28a was found to function in maintained stem cell pluripotency and involved in early embryo nucleolus construction. Until now, few studies got involved in the exact molecular mechanism that affects embryo implantation potential. In this study, the possible function of Wnt/β-catenin-lin28a/let-7 pathway in mouse embryo implantation was studied.

**Methods:**

ICR mouse, Lin28a/Let-7 g transgenic mice (Lin28a-TG/Let-7 g-TG), and implanting dormant mice models were used for the study.

**Results:**

Wnt/β-catenin signaling is essential in embryo implantation, which promotes embryo implantation through directly trigger lin28a expression, thus represses the mir-let-7 family. Lin28a and mir-let-7 both participate in implantation via an inverse function. Lin28a and mir-let-7 participate in embryo implantation through embryonic EMT.

**Conclusions:**

Wnt/β-catenin signaling promotes embryo implantation and accompanying embryonic EMT, which is mediated by directly activate lin28a/let-7 axis.

Video abstract

## Background

Development of the blastocyst to the competent status and orchestrated embryonic EMT that accompany and underlie implantation and embryonic morphogenesis are essential for a successful pregnancy.

The pre-implantation mouse blastocyst is a differentiated, fluid-filled ball that comprises two distinct cell populations, the ICM and surrounded TE [1]. With the exposure of TE cells to the uterine epithelium, implantation and accompanying embryonic EMT begins. EMT is a biologic process that polarized epithelial cells undergo multiple biochemical changes to gain a mesenchymal cell phenotype. The epithelial-like TE cells transform into invasive trophoblast giant cells, known as the yolk sac placenta, to provide a network of anastomotic channels surrounding the embryo and anchor the placenta. This process is called trophectoderm EMT [2]. At the same time, ICM segregates into epiblast (EPI) and primitive endoderm (PE) layers. Then gastrulation EMT initiated when the primitive streak begins to form from EPI, the primitive streak generates the mesendoderm, which subsequently separates to form the mesoderm and the endoderm via an EMT. Those cells remaining in the EPI become ectoderm. Sequential EMTs are needed for the final differentiation of specialized cell types and the formation of the three germ layers that generate all tissue types of the body [3–5]. The exact mechanism that affects embryo implantation competency and EMT is still not clear.

The signaling pathway driven by Wnt/β-catenin seems to be one of the required pathways to achieve blastocyst competency for implantation. Silencing of Wnt/β-catenin signaling in mouse embryo reduced Cdx2, a key transcription factor involved in TE lineage specification, negatively affected the implantation competency of blastocyst [6]. In the post-implantation embryo, Wnt3 deficient mouse embryos have a block in anterior-posterior axis formation, fail to initiate gastrulation, and do not form mesoderm [7]. It was reported active-β-catenin detected in different cells of mouse post-implantation embryo related to primitive streak formation [8], emphasizing the importance of this pathway in embryo development.

MicroRNAs constitute a large family of approximately 21-nucleotide-long, noncoding RNAs. They occupy 5% of the human genome but work as key post-transcriptional regulators of more than 70% of genes through mRNA decay and/or translational inhibition [9]. MicroRNA has been found to function in the reproduction system among oogenesis, spermatogenesis, fertilization and early embryonic development, implantation, and placentation. The dysregulation of miRNA may lead to reproductive disease [10–13]. Our previous microarray data suggested that several members of the let-7 family were up-regulated in the mice dormant blastocysts when compared with activated blastocysts, and Integrin-3βworks as a down-stream target of mir-let-7 responsible for blastocyst adhesion [14]. Besides, we found mir-let-7a reduces human embryo surrogates’ spheroid attachment ability [15].

Lin28a, a natural inhibitor of mir-let-7 family, is a member of a RNA-binding protein (RBP) family conserved in animals [16, 17]. Lin28 family is widely accepted as stem cell maintaining factors that promote stem-cell reprogramming through RNA binding function [18, 19]. Also, lin28a represses the processing of mir-let-7 at both the pri- and pre-miRNA steps, thus affects the translational efficiency of mir-let-7 target genes [20–22]. It has been found that during embryo development, Lin28 enhances the translation of genes important for growth and survival of human embryonic stem cells [23].

In this study, the regulation of mir-let-7 by Wnt/β-catenin-lin28a signaling was investigated, and their function in mouse embryo implantation competency and embryonic EMT were firstly studied.

## Material and method

### Animals and embryo collection

The use of animals was approved by the Committee on Use of Live Animals in Teaching and Research, the University of Hong Kong (Approval no. 3534–4, 4100–16, and 3573–15). Imprinted-coding region (ICR) mice were purchased from Harlan UK Ltd., Bicester, Oxon, UK. We generated a chimeric let-7 g species let-7S21L (let-7 g Stem, mir-21 Loop) in which let-7 g can be induced with doxycycline under the control of the Rosa26 locus (Let-7 g transgenic mice, Let-7 g-TG). Lin28a transgenic (Lin28a-TG) mice was a kind gift from Children’s Hospital Boston and Dana Farber Cancer Institute, Boston, which also can be induced expression by doxycycline. Lin28a-TG and Let-7 g-TG mice were housed with food and water under controlled temperature (24 ± 1 °C), humidity (55 ± 2%), and lighting conditions (daily light period, 0700 to 1900 h). The morning of finding a vaginal plug was designated as Day1 of pregnancy. Embryos at different developmental stages were recovered through flushing the oviduct on the morning of Day 1, 2, or 3 of gestation (1 cell stage embryo, two cell stage embryo, morula) or uterus on Day 4 (blastocyst). Implantation sites were detected on Day 5, Day 6 or Day 7 evening by Chicago blue (1% solution into saline, 2610-05-1, Sigma) via caudal vein injection (20 μl/mice). Transgenic mice were fed with doxycycline hyclate (1 mg/ml, D9891, Sigma) in water from the mating day; for the control group, no doxycycline added. For embryo Wnt activation and inhibition, Wnt activator CHIR99021 (SML1046, Sigma) at 10 μM and Wnt inhibitor mouse DKK (sc-5897-DK) at 1 μg/ml were used.

### Delayed implantation

The whole surgical procedure was completed before 8:00 am. The Day 4 pregnant female mice were anesthetized by phenobarbitone. The operation area was near the abdominal cavity on two sides of the back. After removing body hair, skin, and muscle incision were made to expose the para-ovarian fatty tissue, which, together with the ovary, were cut between the oviduct and the ovary. The muscles layer and the body skin were sewed up separately. The surgical site was disinfected with 70% alcohol after surgery. Meloxicam (2 mg/kg, 71,125–38-7, Sigma) was fed in water to release their pain. Progesterone (P4, 2 mg/mouse, 5341, Calbiochem) was administered on Day 5 and Day 6 of pregnancy by subcutaneous injection to maintain the delayed implantation status [24]. Estradiol (E2, 25 ng/mouse, E8875, Sigma) was administered on Day 7 to activate dormant embryos.

### Embryo immunostaining

Embryos were fixed in 4% paraformaldehyde at room temperature for 30 min. Then washed three times in wash buffer (0.01% triton and 0.01% NP40 in PBS), each for 5 min. Permeabilization in 0.4% triton and 0.4% NP40 in PBS for 15 min at room temperature. Incubation with 1% BSA (A3311, Sigma) for 1 h at room temperature. Embryos were then incubated with primary antibodies against active-β-catenin (1:200, 19,807 s, CST), lin28a (1:200, 8706 s, CST), total β-catenin (1:200, 610,154, BD) at 4 °C overnight. After 4 rounds of wash in wash buffer, each for 10 min, embryos were incubated with anti-rabbit AF488 (A11070, Life Technology Scientific), anti-rabbit AF568 (A11036, Life Technology Scientific), or anti-mouse AF488 (A21202, Life Technology Scientific) secondary antibody at room temperature for 1 h, washed and incubated with DAPI (1: 500 in PBS, 28718-90-3, Sigma) for 5 min. After washing, fluorescence signals were examined under a Zeiss LSM 700 confocal laser microscope (Jena, Germany).

### Immunochemistry (IHC)

Day 5, Day 6, and Day 7 implantation sites were cut, fixed in 4% PFA, embedded in paraffin, and cut from the transverse surface onto glass slides. Deparaffinized slides in xylene for 2 times. Rehydration the slides in gradient alcohol. 3% H_2_O_2_ solution was applied to sections for 30 min to block endogenous peroxidase activity. To unmask the antigenic epitope, slides were boiled in antigen retrieval buffer (S1699, Dako Agilent technologies). Cooled down the slides at room temperature, then rinsed with PBS. Added 1% fetal bovine serum to block the sample at room temperature for 1 h. Applied primary antibody and incubated at 4 °C overnight. Slides were washed with PBS for 4 times. Applied (1:300) biotinylated secondary antibody to the sections and incubated at room temperature for 1 h. Washed with PBS again. Applied ABC buffer (VECTORSTAIN, PK-6100) to the sections and incubated at room temperature for 30 min. Washed slides with PBS. Applied DAB substrate solution (DAKO, K3468) to the sections for color development, Washed slides with PBS. Counterstained slides by Hematoxylin. Dehydrated the tissue slides again through 4 times of alcohol (50, 70, 90 and 100%, 100, 100%), 5 min each. Cleared the tissue slides in three times of xylene and mounting. Antibodies used in this assay were active-β-catenin (1:600, 19,807 s, Cell Signalling), lin28a (1:600, 8706 s, Cell Signalling), E-cadherin (1:600, ab76055, Abcam), N-cadherin (1:600, ab18203, Abcam).

### Embryo qPCR

The High Capacity cDNA RT Kit (4,368,814, Life Technology Scientific) was used to produce cDNA of target miRNAs according to the manufacturer’s instructions. The template of miRNAs was obtained by heating the embryos in 6.2 μl 5% NP40 (6507, Sigma) at 95 °C for 10 min. 10 embryos were pooled in each sample. The miRNA reverse transcription primers and probes for mature let-7a-5p (000377, Thermo Fisher Scientific), let-7 g-5p (002282, Thermo Fisher Scientific), U6snRNA (001973, Thermo Fisher Scientific) were used. The reverse transcription condition was 16 °C for 30 min, 42 °C for 30 min, 4 °C for 5 min. The procedure of qPCR was 50 °C for 2 min, 95 °C for 10 min, 95 °C for 10 s, and 60 °C for 1 min.

### Mouse primary endometrium (ME) cell isolation

Pregnant Day 4 mouse uteri were cut from mouse abdominal cavity, removed fat tissues, and washed in PBS. Cut longitudinally and digested in 0.5% trypsin (215,240, Becton Dickinson) and DNase I (180,470, Life Technology Scientific) at 4 °C for 90 min and then at 37 °C for 30 min. Tissues were then transferred to complete DMEM/F-12 (11,320,033, Thermo Fisher) and shaken gently for 30 s, three times. The dispersed luminal epithelium and some glandular epithelium in the upper cell suspension were collected and centrifuged at 1500 rpm for 5 min, then washed by centrifugation in complete DMEM-F12 for another 2 times. The recovered epithelial cells were seeded or collected.

### Chromatin immunoprecipitation (CHIP)

The procedure was according to the manufacturer (185,913, Abcam). The PCR primers used were as follows: Site 1 forward: GGGGTTGGGTCATTGTCTTT, Reverse: CTCGGTACCAAGCTTGCG; Site 2 forward: GCACTTTCTCTTTTCTAACTGGG, Reverse: GGAGTTCTGGACACTGGGAA, Site3 forward: GCTTACCTGGGGCTAACTTT, Reverse: ATCTGAATGTCTGCCTCCCA; Site 4 forward: GAGCGCATTTTAGGGTGGTT, Reverse: TTCCTCCCTCCAAACCTGTG; Site5 forward: CAGTCCTGTTCCTCCACCAG, Reverse: CATCCACTGCTGAGAGAGGG; Published site 1 forward: GTGCGGGGGAAGATGTAGC, Reverse: CAAACTGCTGGTTGGACACG; Published site 2 forward: GTGGTTTGTGTTTCTGATTGGC, Reverse: GAGTACCCCGGAATTTGAGATC. The PCR procedure was according to Taq 2X Master Mix (M0270, NEB), that was 95 °C for 30 s, 95 °C for 30 s, 45–68 °C for 1 min, 68 °C 1 min per kb, 68 °C for 5 min, 35 cycles from the second step to the fourth step.

### In vitro embryo attachment and outgrowth

Embryos were retrieved from pregnant Day 4 mouse uteri. The zona pellucida of blastocysts was removed by acid tyrode (CaCl_2_ 265 g mg, MgCl_2_ 0.0469 g, KCl 0.2 g, NaCl 8 g, NaH_2_PO_4_ 0.05 g to 1 l and adjust pH to 2). Embryos were then put on to Day 4 ME monolayer. To determine attachment, the plate was shaken at a rate of one rotation/second on an orbital shaker for 20 s after 24 h culture. Blastocysts that remained at the same location were designated as attached blastocysts. At 48 h, outgrowth was identified as the area covered by trophoblastic monolayer around the embryo.

### Embryo electro-transfection

Blastocysts were retrieved from pregnant Day 4 mouse uteri. Let-7a inhibitor (AM10050, Thermo Fisher), lin28a siRNA (SC-106990, Santa Cruze), Scramble control siRNA (SC-37007, Santa Cruze) were electro-transfected into blastocysts in a flat electrode (1 mm gap between electrodes) in 20 μl of Hepes-buffered saline (150 mM NaCl, 20 mM HEPES) by two sets of 3 electric pulses of 1 ms at 30 V using a 830 Electro Square Porator (BTX Inc., CA). Following electro-transfection, embryos were cultured in fresh M16 medium (M7292, Sigma), and waited for recovery.

### Statistical analysis

All the images of confocal microscopy were representatives of at least three independent experiments. qPCR assays were performed in triplicate, and each experiment was repeated for at least three times. Attachment/outgrowth assay has been done at least four times. Data shown were presented as means ± sd. Differences were considered statistically significant at *p* < 0.05 using one way ANOVA.

## Result

### Wnt signaling is essential in embryo implantation

**Fig. 1:**
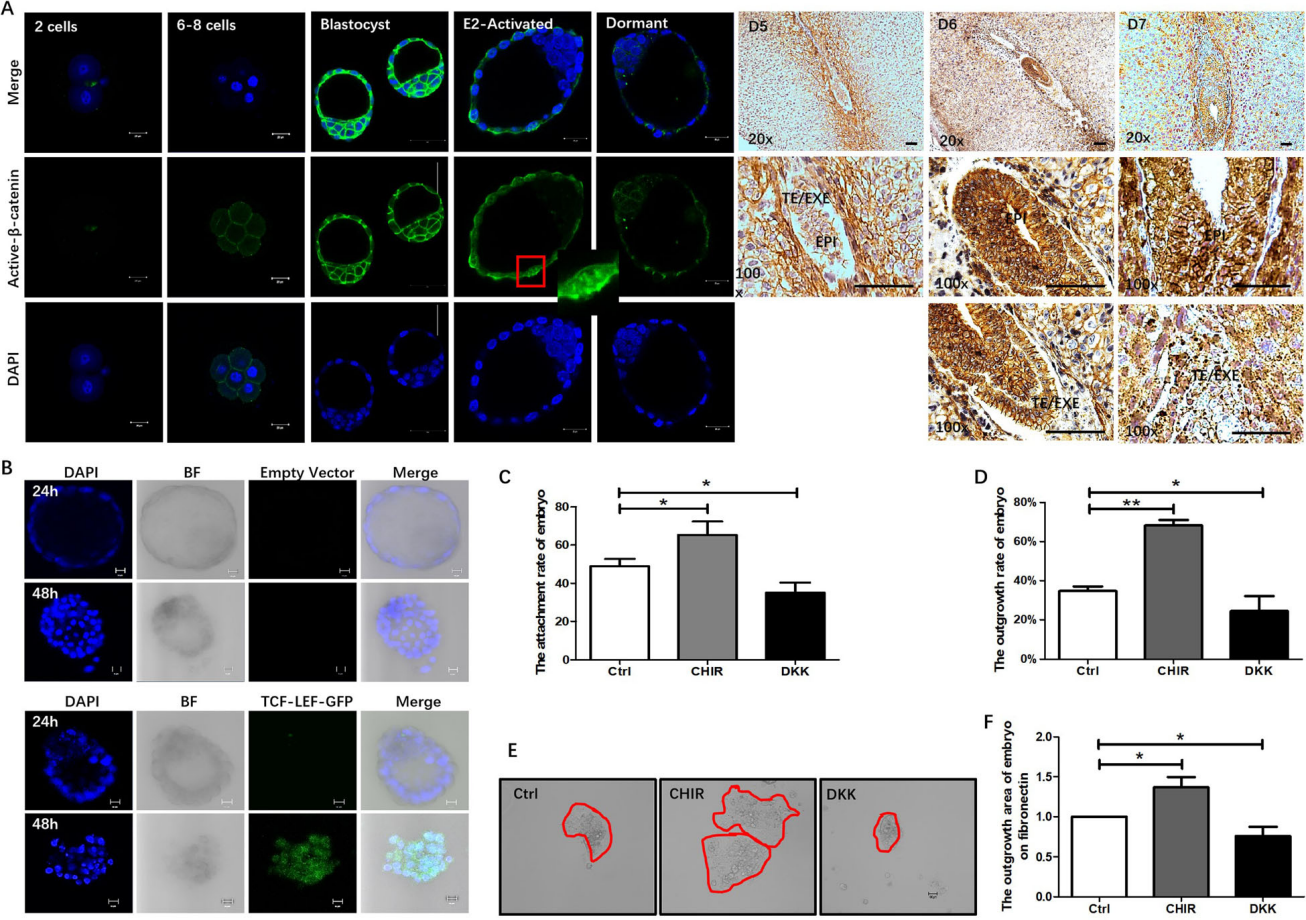
Wnt signaling is essential in embryo implantation. **a** Location of active-β-catenin in pre-implantation, dormant, estrogen-activated, and post-implantation Day 5 to Day 7 embryo. Active-β-catenin is in green, nuclei is in blue, pictures were captured under 40 x objective in confocal 700, scale bar was shown as 20 μm or 40 μm according to different zoom area. TE: trophectoderm, EXE: extra-embryonic ectoderm, EPI: epiblast. IHC scale bar 30 μm at 20X, 150 μm at 100 X. **b** Expression of Wnt-stimulated GFP during embryo outgrowth on fibronectin. **c** In vitro attachment rate of embryos with Wnt activation or inhibition for 24 h. **d**, **e**, **f** The outgrowth rate, status, area of embryos after Wnt activation, and inhibition for 48 h, bar = 100 μm. Data are means ± s.d. (*N* = 3–5), *p* < 0.05 “*”, *p* < 0.01 “**”

Previous researches revealed that Wnt/β-catenin signaling is necessary for embryo implantation competency [6]. Mouse embryo implantation happens at blastocyst stage in Day 4.5. In the pre-implantation embryo, active-β-catenin mainly localized on the cellular membrane and increased from 2-cell stage embryos to blastocysts (Fig. 1a). In E2 activated implanting competent embryos, active-β-catenin was observed in both membranes, and the nucleus of the trophectoderm compares with faint immunoactivity only in the membrane of implanting dormant embryos (Fig. 1a). Compare with pre-implantation embryos, active-β-catenin is not only appeared on cellular membrane but also on cytoplasm and nucleus of whole embryo from Day 6 onward, suggesting that Wnt/β-catenin is activated in post-implantation embryos, similar with what happened in E2 activated embryos (Fig. 1a).

To further demonstrate Wnt/β-catenin is necessary for embryo implantation. p-GMT-TCF/LEF-GFP vector, which produces GFP protein upon activation of the canonical Wnt/β-catenin pathway, was electro-transfected into mouse blastocysts. GFP fluorescence was seen automatically expressed in the whole embryo during outgrowth at 48 h, confirming that the pathway was stimulated within this period (Fig. 1b).

The function of Wnt in embryo implantation competency was further studied. Blastocysts were cultured on fibronectin to exclude the effect of the uterine epithelium. Embryos were cultured with Wnt activator CHIR99021 or inhibitor DKK containing M16 medium for 24 h to count attachment and 48 h to observe outgrowth. Wnt stimulation increased embryo attachment (Fig. 1c). At 48 h, more embryos in the Wnt activation group exhibited much evident outgrowth when compared with control, while embryo in the DKK treated group shown decreased outgrowth rate (Fig. 1d). The outgrowth status and outgrowth areas of embryos at 48 h were shown in Fig. 1e and in Fig. 1f, respectively. Similar to the outgrowth rate, Wnt activation enlarged outgrowth area while Wnt inhibition prohibited such expanding ability. Temporal and spatial expression of Wnt ligands in mouse blastocyst has been reported [25, 26], indicating an autocrine manner of embryonic Wnt activation. In addition, the paracrine manner of secrete Wnt ligands from uterus has been testified by detecting Wnt ligands and receptors in peri-implantation mouse uteri and blastocysts in Fig. S1. In summary, Wnt signaling is essential for embryo implantation competency.

### Repression of let-7a/g by Wnt/β-catenin pathway in the mouse embryo

**Fig. 2:**
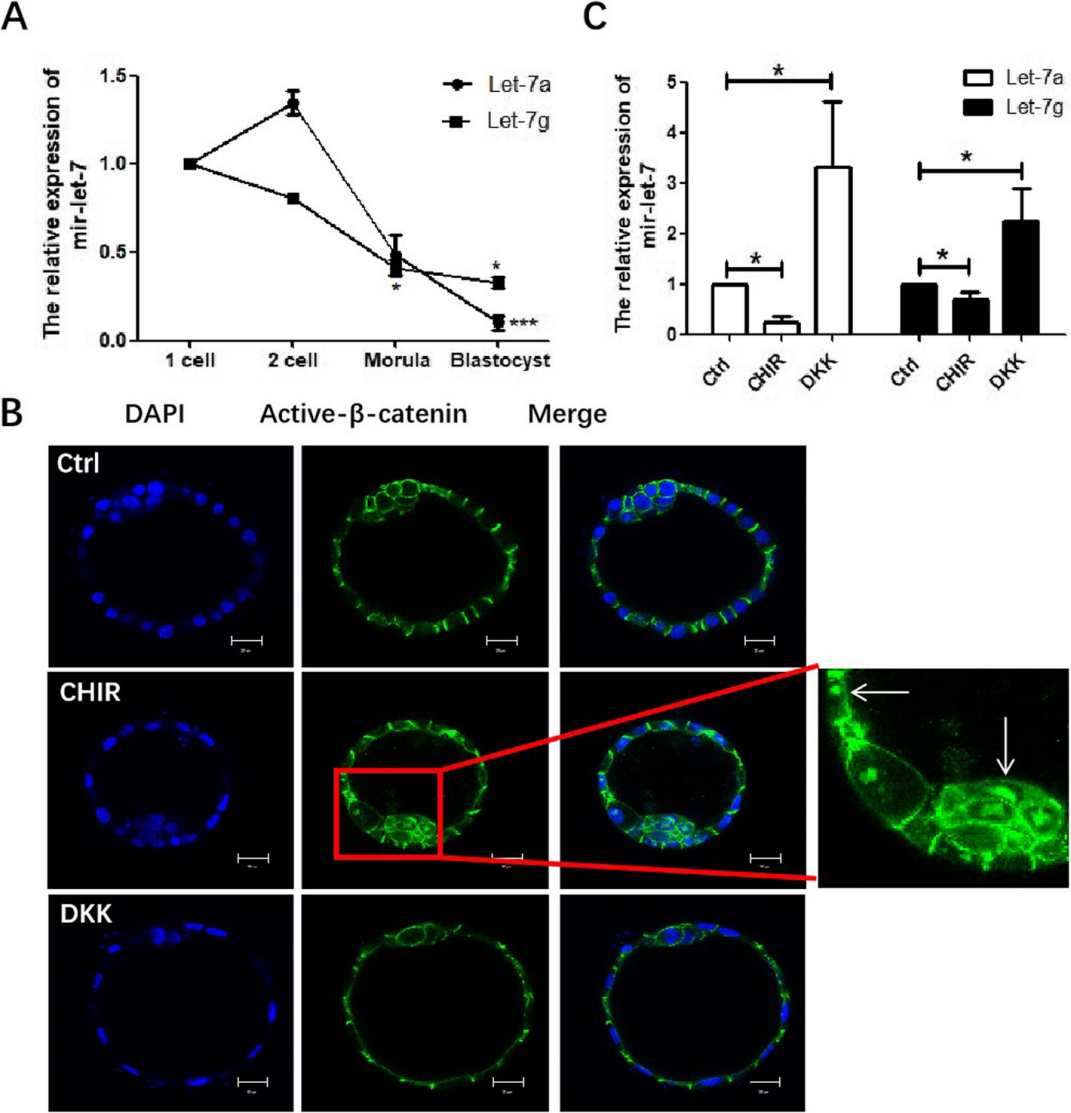
Repression of let-7a/g by Wnt/β-catenin pathway in mouse embryo. **a** Expression of mature let-7a/g in pre-implantation embryo from 2 cell stage to blastocyst, mir-16 was used as internal control. **b** The location of active-β-catenin in embryo upon Wnt activation or inhibition. CHIR99021(10 μM) and DKK (1 μg/ml) treated for 24 h. (Arrow: nuclear active-β-catenin) (**c**) qPCR analysis of the mature let-7a/g in blastocysts with Wnt activation by CHIR99021 or Wnt inhibition by DKK for 24 h. Data are means ± s.d. (*N* = 5), bar = 20 μm, *p* < 0.05 “*”, *p* < 0.01 “**”

Several observations suggest that miRNAs are involved in early pre-implantation embryo development and implantation process [14, 27]. Our previous data compare miRNA expression between delay implanting dormant and E2 activated embryos, found a significant decrease in the mir-let-7 family members (including let-7a and let-7 g) in the activated embryos [14]. Therefore, the change in let-7 expression in mouse pre-implantation embryos was detected. Mir-let-7a and let-7 g were chosen as representative of the family. qPCR detection of the mature let-7a/g shown that the expression decreased more than 50% from the zygote stage to blastocyst, with let-7a decreased at blastocyst stage while let-7 g declined from morula (Fig. 2a), confirming that the mature let-7 expression was suppressed before embryo implantation.

Pathway analysis suggested that Wnt signaling was potentially associated with the differential miRNAs expression between activated and dormant embryos [14]. Wnt activator CHIR99021 and Wnt inhibitor DKK were used to treat blastocysts and cultured for 24 h in M16 medium to determine if the perturbation of Wnt signaling would affect let-7 expression. With Wnt stimulation, active-β-catenin goes into the cell nucleus in both ICM and TE cells (Arrow indicated) (Fig. 2b), the corresponding let-7a/g level was decreased (Fig. 2c). DKK treatment decreased active-β-catenin in the embryo but up-regulate let-7a/g (Fig. 2b, c). Taken together, the let-7 family member is under negative regulation of Wnt signaling in the embryo.

### Mir-let-7 family member decreases embryo implantation competency

**Fig. 3:**
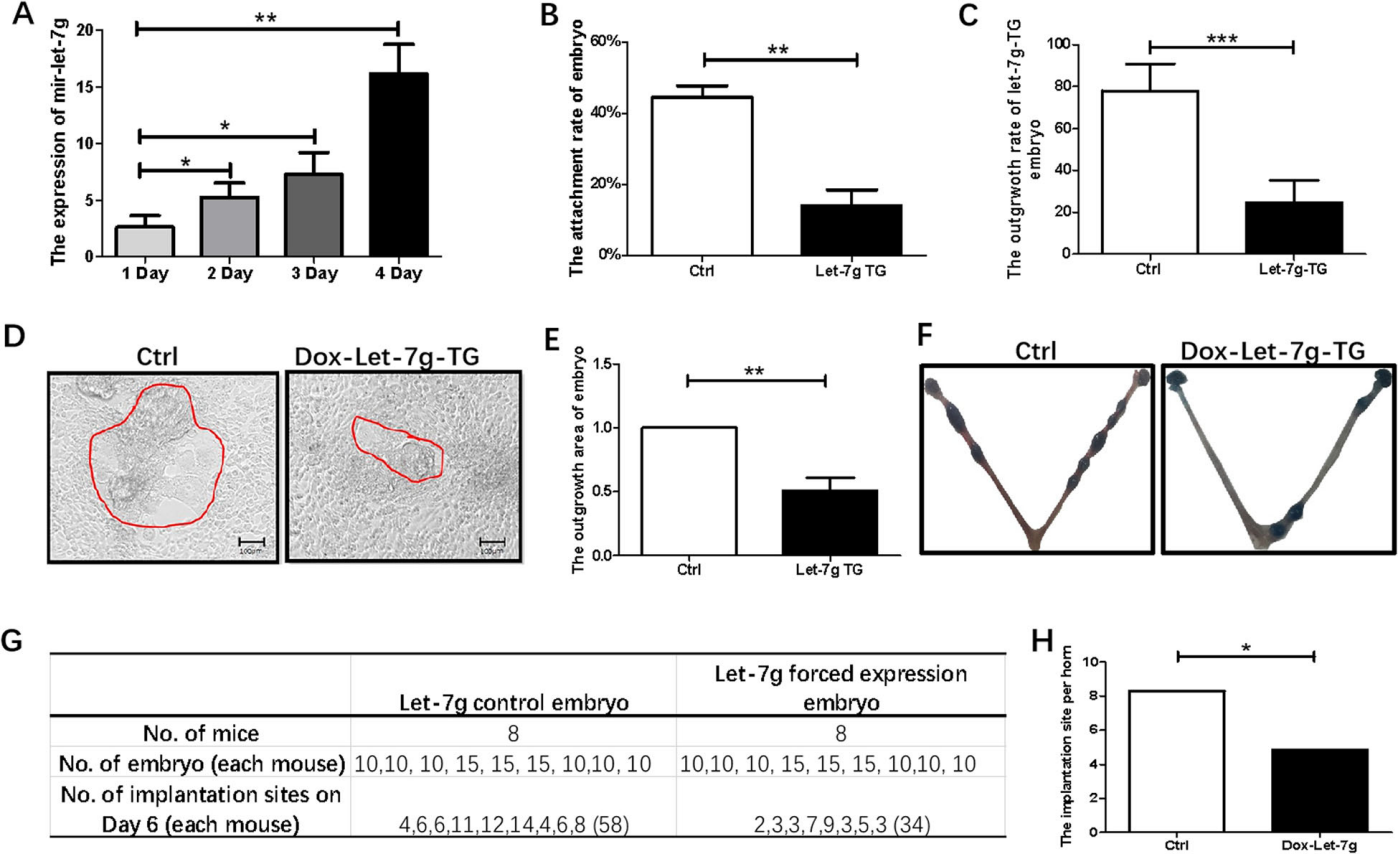
Mir-let-7 family decreases embryo implantation competency. **a** The increasing level of let-7 g in let-7 g-TG mice embryo after doxycycline fed for 1/2/3/4 days. **b** The attachment rate of let-7 g-TG mice embryo. **c** In vitro outgrowth rate of let-7 g-TG mice embryo. **d** The outgrowth status of let-7 g-TG embryo. **e** Relative outgrowth area in let-7 g-TG embryo. **f**, **g**, **h** Implantation site of let-7 g overexpressed or control embryo transferred pseudopregnant mice at Day 6. Data are means ± s.d. (*N* = 3–5), bar = 100 μm, *p* < 0.05 “*”, *p* < 0.01 “**”

Our lab occasionally owns let-7 g-TG mice. As let-7 family members contain the same core sequence, function similarly but location in different chromosomes. Let-7 g-TG mice model was used to study the function of let-7 family in embryo implantation. After 4 days of induction, the let-7 g level in blastocysts was elevated by more than 15 times relative to the control blastocysts without induction (Fig. 3a). When let-7 g overexpressed blastocysts were put onto normal ME in the presence of doxycycline, only 13.33% embryos attached compared with control of 46.66% after 48 h of co-culture (Fig. 3b). At 72 h, let-7 g overexpressed embryos had a much lower outgrowth rate (Fig. 3c) and reduced outgrowth area (Fig. 3d, e) than the control without doxycycline treatment.

For the in vivo implantation model, 10–15 Let-7 g-TG blastocysts that have been fed with doxycycline for 4 days and control blastocysts that have a normal level of let-7 g were transferred into each pseudo-pregnant mice on Day 4. The number of implantation sites was determined on Day 6 of pregnancy (Fig. 3f, g, h). The implantation site in let-7 g forced-expressed embryo was evidently decreased when compared with those transferred with normal embryos.

In summary, these results demonstrate that a high level of let-7 family members inhibits embryo implantation competency.

### Lin28a is directly up-regulated by Wnt/β-catenin and participates in implantation

**Fig. 4:**
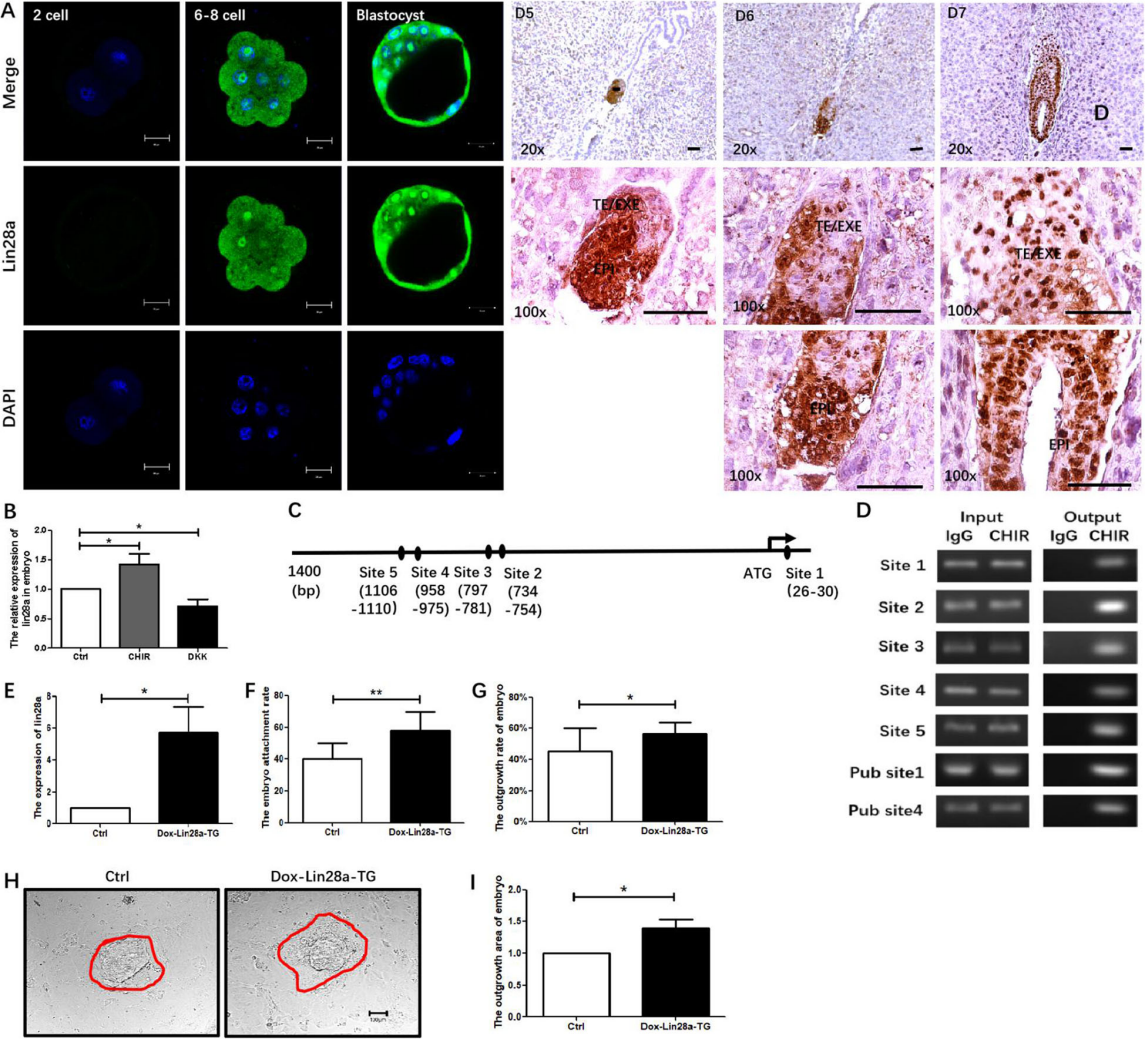
Lin28a is directly up-regulated by Wnt/β-catenin and participates in implantation. **a** Lin28a expression in pre-and post-implantation Day 5 to Day 7 mouse embryo. Lin28a is in green, nuclei is in blue, pictures were captured under 40X objective in confocal 700, scale bar was shown as 20 μm. TE: trophectoderm, EXE: extra-embryonic ectoderm, EPI: epiblast. IHC scale bar 30 μm at 20X, 150 μm at 100 X. **b** Lin28a mRNA level in Wnt activation and inhibition embryo. **c**, **d** The PCR result of CHIP binding site. 5 sites have been newly detected, 2 sites were published sites (Pub 1 and 4). **e** The relative expression of lin28a mRNA in lin28a-TG mice embryos after doxycycline induction for 4 days. **f**, **g** In vitro attachment/outgrowth rate of lin28a-TG mouse embryos on ME cells at 24 h. **h**, **i** The outgrowth status and area of lin28a-TG embryos. Data are means ± s.d. (*N* = 3–5), bar = 100 μm in **h**, *p* < 0.05 “*”, *p* < 0.01 “**”

Result in Fig. 2 demonstrated that Wnt signaling modulated let-7 expression. Lin28a is a well-known natural inhibitor of let-7 family [16, 28]. It was hypothesized that lin28a functioned downstream of Wnt signaling to repress let-7. Therefore, lin28a was determined in the pre-implantation and post-implantation Day 5 to Day 7 embryos. The expression of lin28a was like that of active-β-catenin that was up-regulated from 2 cell stage to blastocyst (Fig. 4a). From post-implantation Day 5 onward, lin28a expressed in the cytoplasm and the nucleus in both the TE and the EPI. On Day 5 and Day 6, more lin28a was detected in the EPI than the TE. On Day 7, lin28a expressed in the whole embryo (Fig. 4a).

At the mRNA level, lin28a mRNA increased upon activation and decreased after inhibition of Wnt (Fig. 4b). These results demonstrated that lin28a was positively regulated by Wnt/β-catenin in embryos. To further examine whether β-catenin in canonical Wnt pathway activates lin28a transcription by direct binding to its promoter as a co-activator of LEF/TCF, CHIP assay was performed in mouse trophoblastic stem (mTS) cells, which highly express lin28a as lin28a is a stem cell maintaining factor [18]. It revealed β-catenin specifically binding to lin28a promoter at five sites in a sequence range upstream of lin28a TSS 1337 bp and downstream of TSS 84 bp. Besides, two sites that have been published also can be detected in mTS cells (Fig. 4c, d, Pub site1, and 4) [29, 30]. Further, the function of lin28a in embryo implantation competency was detected. The level of lin28a in doxycycline induced Lin28a-TG blastocysts was confirmed by qPCR (Fig. 4e). Lin28a overexpression elevated embryo attachment rate from 41.31 to 53.34% (Fig. 4f). The outgrowth rate increased from 45.67 to 56.31% at 48 h (Fig. 4g). With lin28a overexpression, the embryo outgrowth area was evidently raised (Fig. 4h, i). Collectively, these results demonstrate that lin28a is direct positively regulated by Wnt/β-catenin and important for embryo implantation.

### The function of Wnt in embryo implantation is mediated through lin28a/let-7a axis

**Fig. 5:**
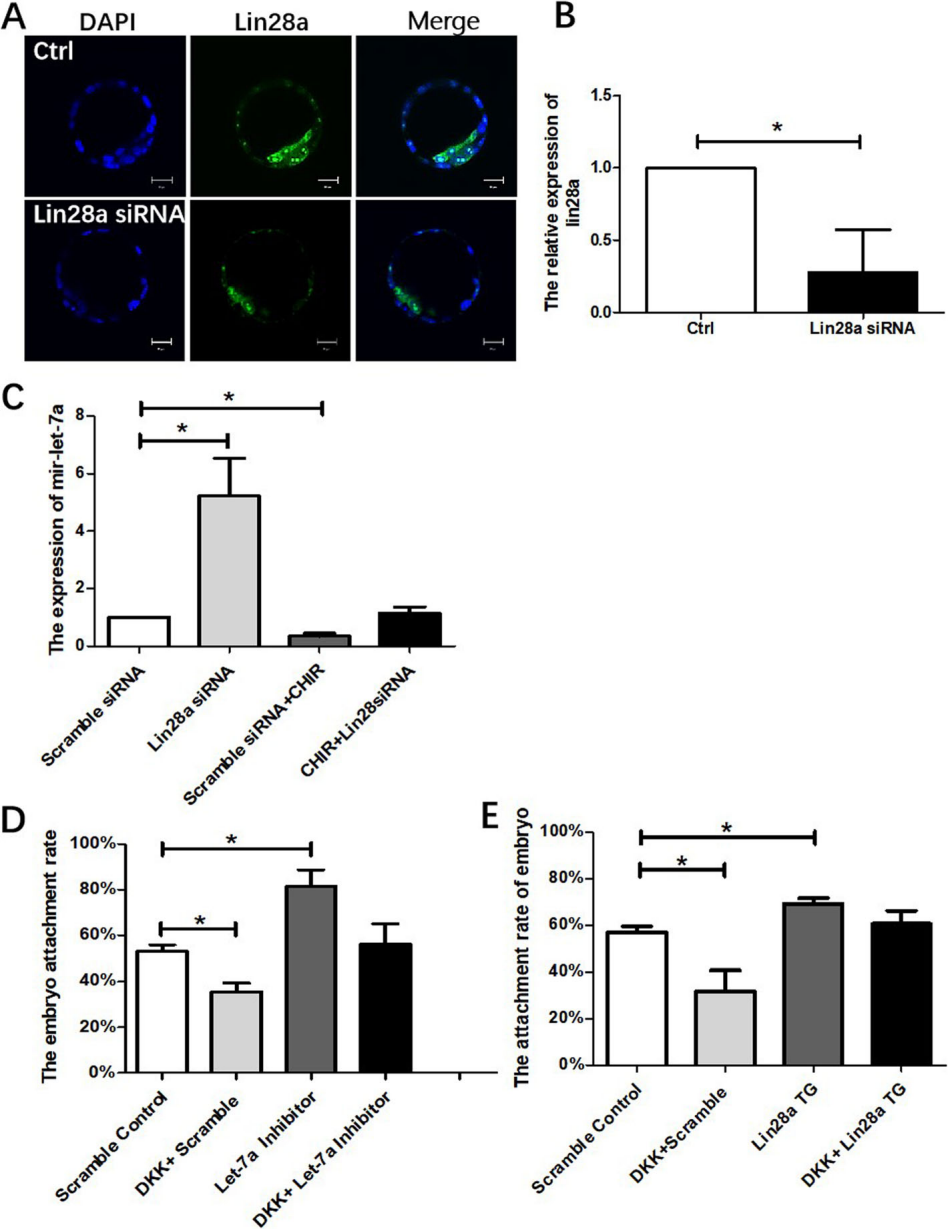
The function of Wnt in embryo implantation is mediated through lin28a/let-7. **a** The efficiency of lin28a siRNA knockdown in blastocysts by immunostaining. **b** The relative lin28a mRNA level in lin28a siRNA knockdown embryo. **c** The relative expression of let-7a in lin28a knockdown but Wnt activation blastocyst. **d** Embryo attachment rate on fibronectin when embryo Wnt was inhibited and/or let7a was knockdown. **e** Embryo attachment rate on fibronectin when embryo Wnt was inhibited by DKK and/or lin28a was overexpressed (lin28a-TG). Data are means ± s.d. (*N* = 3–5), bar = 20 μm, *p* < 0.05 “*”, *p* < 0.01 “**”

To verify whether the regulation of Wnt/β-catenin on let-7 was through lin28a in mouse embryos. Blastocysts were electro-transfected with scrambled control siRNA and lin28a siRNA respectively and cultured for 24 h in M16 medium. The efficiency of lin28a knockdown was confirmed by immunofluorescence staining and qPCR, respectively (Fig. 5a, b). The ICM in the lin28a-siRNA group is less than control may ascribe to the function of cell proliferation was inhibited by lin28a knockdown [18, 19]. Besides, combined Wnt activation and lin28a knockdown, the level of let-7a restored to that of the control electro-transfected with scrambled siRNA and without Wnt stimulation (Fig. 5c). These outcomes suggest that lin28a mediates the repression action of Wnt on let-7.

It was further hypothesized that Wnt signaling facilitates embryo implantation via lin28a/let-7 axis in the process of implantation. Mouse blastocysts were electro-transfected with let-7a inhibitor or scramble siRNA in the presence or absence of Wnt inhibitor DKK. The treated embryos were cultured on the fibronectin-coated plate in M16 medium for 24 h. DKK alone decreased embryo attachment on fibronectin, while let-7a inhibitor alone increased embryo attachment. Co-treatment of the embryo with DKK and let-7a inhibitor abolished the suppressive effect of Wnt inhibition on embryo attachment (Fig. 5d), suggesting that let-7a mediated the action of Wnt on embryo implantation. A similar experiment was done with lin28a overexpression in lin28a-TG embryo with DKK treatment (Fig. 5e). With Wnt inhibition, the embryo attachment rate was decreased significantly, while lin28a overexpression increased the attachment rate. When DKK was applied to lin28a overexpressed embryos, the attachment rate rescued to the control level. Taken together, lin28a/let-7a signaling mediates the effects of Wnt on embryo implantation.

### Let-7 family members disturb embryonic EMT

**Fig. 6:**
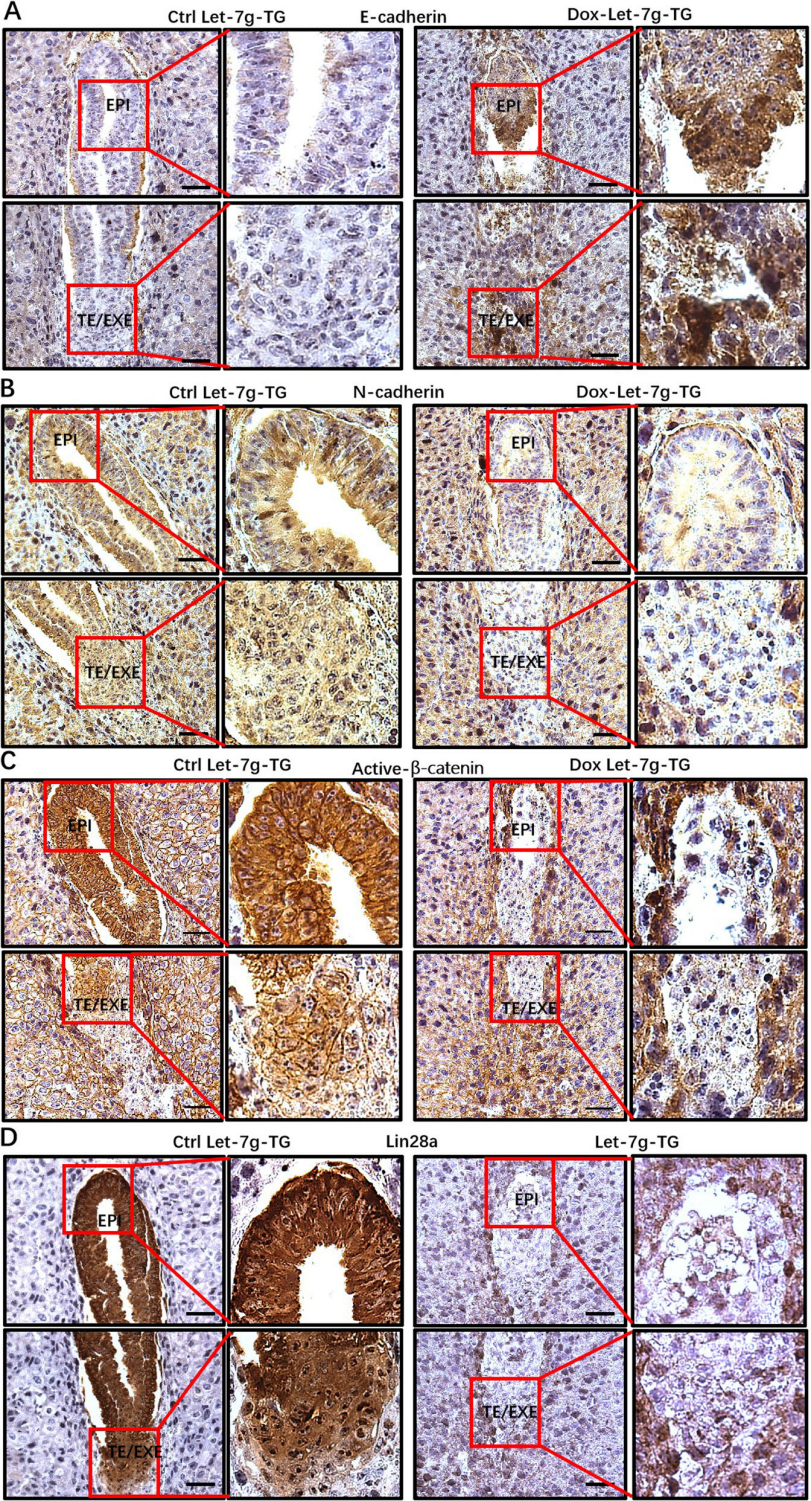
Let-7 family members disturb embryonic EMT. **a** Immunochemistry staining of E-cadherin, **b** N-cadherin, **c** Active-β-catenin, **d** Lin28a in control let-7 g and let-7 g overexpressed embryo (Let-7 g-TG) after embryo transfer. EPI: epiblast. TE: trophectoderm. EXE: extra-embryonic ectoderm. Scale bar: 60 μm

Embryonic EMT participates in implantation, placentation, and organ development, which is essential for a successful pregnancy [4, 31, 32]. To observe whether the adverse effect of the let-7 family member on embryo implantation was because of disturb EMT process. Blastocysts with or without let-7 g overexpression were transferred to pseudopregnant normal Day 4 female mouse uteri. Implantation sites were detected on Day 6 by Chicago blue caudal vein injection, fixed and sectioned for IHC staining. As shown in Fig. 6a, epithelial marker E-cadherin was stronger in let-7 g-TG embryo. In contrast, mesenchymal marker N-cadherin expressed in the whole uterus as the majority of the uterine cells at this stage were mesenchymal cells, which was significantly less in the let-7 g-TG embryos relative to untreated control (Fig. 6b), indicating impairment of EMT. The expression of active-β-catenin (Fig. 6c) and lin28a (Fig. 6d) were higher in the control embryos than the let-7 g overexpressed embryos. These outcomes reveal overexpressed let-7 g in embryo disturb embryo implantation via impairing EMT process. In addition, the in vitro embryonic EMT process was also promoted by lin28a (Fig. S2), while attenuated by the let-7 g (Fig. S3).

## Discussion

Active-β-catenin has been reported expressed in the nucleus of mouse embryos from 1 cell to blastocyst [6]. However, nuclear active-β-catenin could not be detected in the pre-implantation stages in this research even with the use of the same reported antibody or other antibodies claimed against active-β-catenin. Instead, active-β-catenin localized to the cellular membrane, despite there was an increasing trend in the pre-implantation stage of pregnancy. From Day 6 onward, active-β-catenin was detected in the cell membrane, cytoplasm, and nucleus. The finding is consistent with the current understanding of Wnt signaling in embryo development and implantation, that is, Wnt signaling is not necessary for blastocyst formation, which is supported by (1) DKK treatment has no apparent adverse effect on embryo develop from 2 cell stage to blastocysts [6, 33, 34]. (2) β-Catenin-null embryos (β-cat −/−) can develop normally until Day 6.5 [35]. (3) Truncated β-catenin in the embryo doesn’t affect embryo development to blastocyst in vitro [36]. Besides, it is reported that β-catenin forms adhesion junction with E-cadherin soon on the cellular membrane after being synthesized [37]. E-cadherin sequesters β-catenin onto the cellular membrane, without which β-catenin can go into the nucleus [36, 38, 39]. I have detected pre-implantation embryo E-cadherin, it was indeed on the cellular membrane (data not shown). Taken together, firstly, Wnt signaling is not a necessary condition for blastocyst formation; Secondly, β-catenin is sequestered by E-cadherin on cellular membrane. These results together explain the pre-implantation membrane staining of active-β-catenin in this finding.

Though active-β-catenin mediated canonical Wnt signaling is not required for pre-implantation embryo development, silencing of Wnt/β-catenin signaling in pre-implantation embryo reduces Cdx2, a key transcription factor involves in TE lineage specification, and considerably reduced implantation rate upon transfer to pseudo-pregnant recipients, and the Wnt downstream target c-Myc, which has been shown to be crucial for pre-implantation embryo development, is also down-regulated in blastocyst TE [6]. Similarly, in porcine parthenogenetic (PA) embryos, inhibition of Wnt signaling doesn’t alter the blastocyst formation rate. However, the hatching rate is decreased, and TE development is blocked. In addition, with the activation of Wnt signaling, PA blastocysts hatching improved dramatically [40]. These findings together suggest that Wnt signaling is required for embryo implantation competency .

Embryonic EMT during implantation is designed as type I EMT [3]. In our finding, active-β-catenin goes into cytoplasm and nucleus from Day 6.5 onward, stimulation Wnt signaling in embryo improves implantation capacity by strengthening embryo attachment and outgrowth ability. Besides, in let-7 g overexpressed, implantation competency weaken embryo, the post-implantation EMT process was disturbed with an impairment of Wnt/β-catenin signaling in vivo. In vitro, the embryonic EMT process was also promoted by lin28a but attenuated by the let-7 g. These evidences together provide support for the role of the canonical Wnt/β-catenin pathway in embryo implantation and accompanying EMT.

In the present study, lin28a locates in both cytoplasm and nucleus, especially in the nucleolus of mouse embryos (Fig. 4). Lin28a has been reported to work in both the cytoplasm and the nucleus; it recruits a TUTase to block the processing of let-7 precursors by Dicer in the former, while sequesters primary let-7 transcripts inhibiting their processing by the microprocessor in a TUTase independent manner in the latter [19, 41]. SET7/9-mediated methylation of lin28a at lysine 135, a region homologous with the NoLS (Nucleolar Localization Signal) of its homologous family member lin28b, increases lin28a stability, promotes localization of the molecule in the nucleus and multimerization with mir-let-7 [42]. Besides, lin28a is an essential factor of nucleolar-genesis during early mouse embryonic development [43]. These evidences combine with the present findings of high expression of lin28a in pre and post-implantation embryo and its function in promoting EMT suggest lin28a is important for implantation.

In breast cancer, Wnt/β-catenin inhibits breast cancer stem cell expansion through direct binding to lin28a promoter and inhibits let-7a [30]. Besides, the direct regulation of Wnt to lin28 was also found in the adult mammalian retina. CHIP assay reveals that β-catenin binding to two distinct sites in the proximal region of lin28a and lin28b promoters. This binding is critical for lin28 family transcriptional activation [29]. Interestingly, the two β-catenin binding sites in the two types of research are different, suggesting that distinct β-catenin binding sites may be used to drive lin28 transcription in different tissues. A similar situation is found in this research: a total of five β-catenin binding sites were detected by CHIP assay in mouse trophoblast stem cells. Besides, the two binding sites that have been reported also can be detected [29]. It is possible that the already published four sites were included in the 5-binding sites in this finding; however, it is undeterminable as there is no detail information for those sites find in their studies.

Let-7a/g were decreased suddenly in the pre-implantation embryo. Overexpressed let-7 g in let-7 g-TG mice embryo decreased embryo implantation ability. Integrin-3β has been proved as a down-stream target of mir-let-7 in our previous study [14]. Integrins are a family of heterodimeric (α/β) transmembrane molecules that responsible for blastocyst adhesion through activation of phosphoinositide-specific phospholipase C leading to initiation of phosphoinositide signaling [44]. Integrin αvβ3 has been detected on the apical surface of the TE of blastocysts and mediates primary trophoblast adhesion and migration [45]. Thus, decreased integrin-3β in the high let-7 g embryo may be a possible reason for impaired embryo implantation ability.

There is only one publication mentioned that the promoter activity of mir-let-7a2 in the breast cancer cell line could be downregulated by Wnt activator lithium chloride in luciferase assay [46]. It means Wnt could suppress let-7a2 transcriptional activity through repressing its promoter, but whether this is a direct effect is not known and needs further study.

Wnt signaling and the mesenchymal marker were decreased in let-7 g overexpressed embryonic cells. It means let-7 g inhibit EMT process in the embryo. It has been reported overexpressed let-7b activating epithelial marker E-cadherin and attenuating mesenchymal marker Vimentin, EMT transcriptional factor Twist as well as β-catenin in malignant pleural mesothelioma [47]. In this research, with let-7 g overexpression, EMT process in post-implantation embryonic development was blocked.

## Conclusions

In this study, current understanding is Wnt/β-catenin is essential for proper embryo implantation, and EMT, which function via directly binding to lin28a promoter to activate its transcription, thus represses mir-let-7 biogenesis. Lin28a and mir-let-7 family inversely function in embryo implantation competency and EMT under the regulation of Wnt signaling. In conclusion, our studies first shed light on Wnt/β-catenin-lin28a/let-7a pathway in embryo implantation and accompanying EMT.

## Supplementary information

**Additional file 1.**

**Additional file 2.**

**Additional file 3.**

## Data Availability

The datasets used and analysed during the current study are available from the corresponding author on reasonable request.

## Abbreviations

ICM: Inner cell mass
TE: Trophectoderm
EMT: Epithelial-mesenchymal transition
Lin28aTG/Let-7 gTG: Lin28a/Let-7 g transgenic mice
EPI: Epiblast
ICR: Imprinted-coding region
ME: Mouse primary endometrium
mTS: Trophoblastic stem
