# Supplementary figures and images for: The role of Wnt/β-catenin-lin28a/let-7 axis in embryo implantation competency and epithelial-mesenchymal transition (EMT)
Source: Cell Commun Signal. 2020 Jul 11;18:108. doi: 10.1186/s12964-020-00562-5 (PMC7353806; doi:10.1186/s12964-020-00562-5)

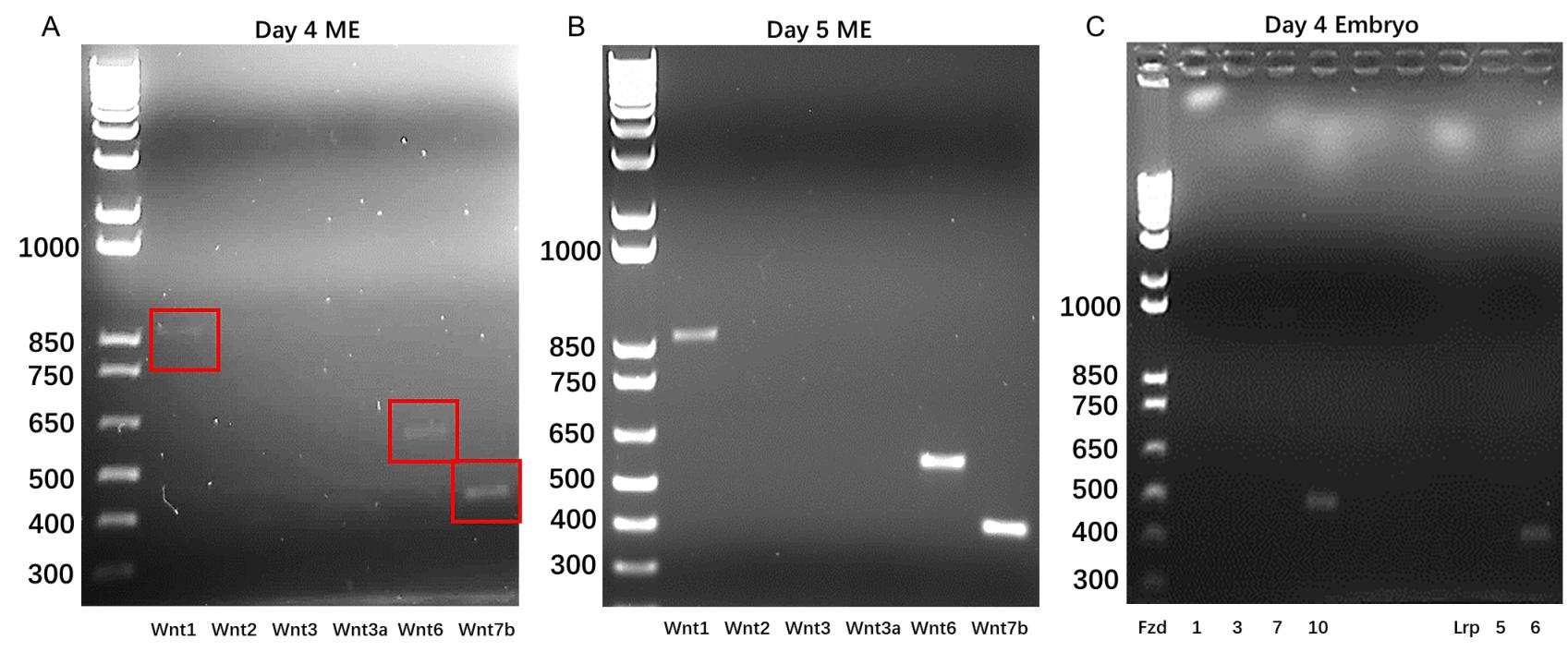

Supplement: Supplementary file 2 — Additional file 1. [file 12964_2020_562_MOESM2_ESM.jpg]

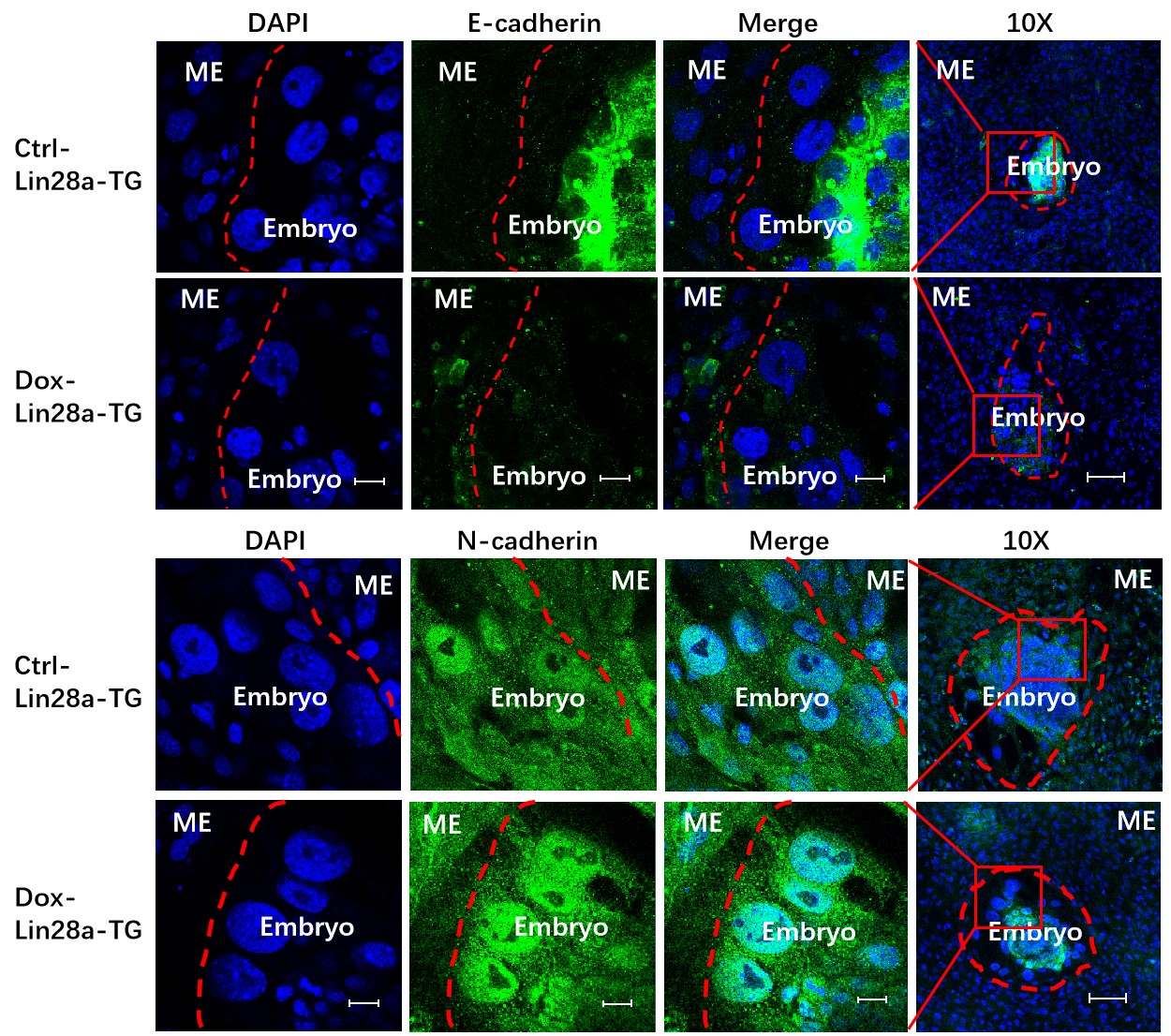

Supplement: Supplementary file 3 — Additional file 2. [file 12964_2020_562_MOESM3_ESM.jpg]

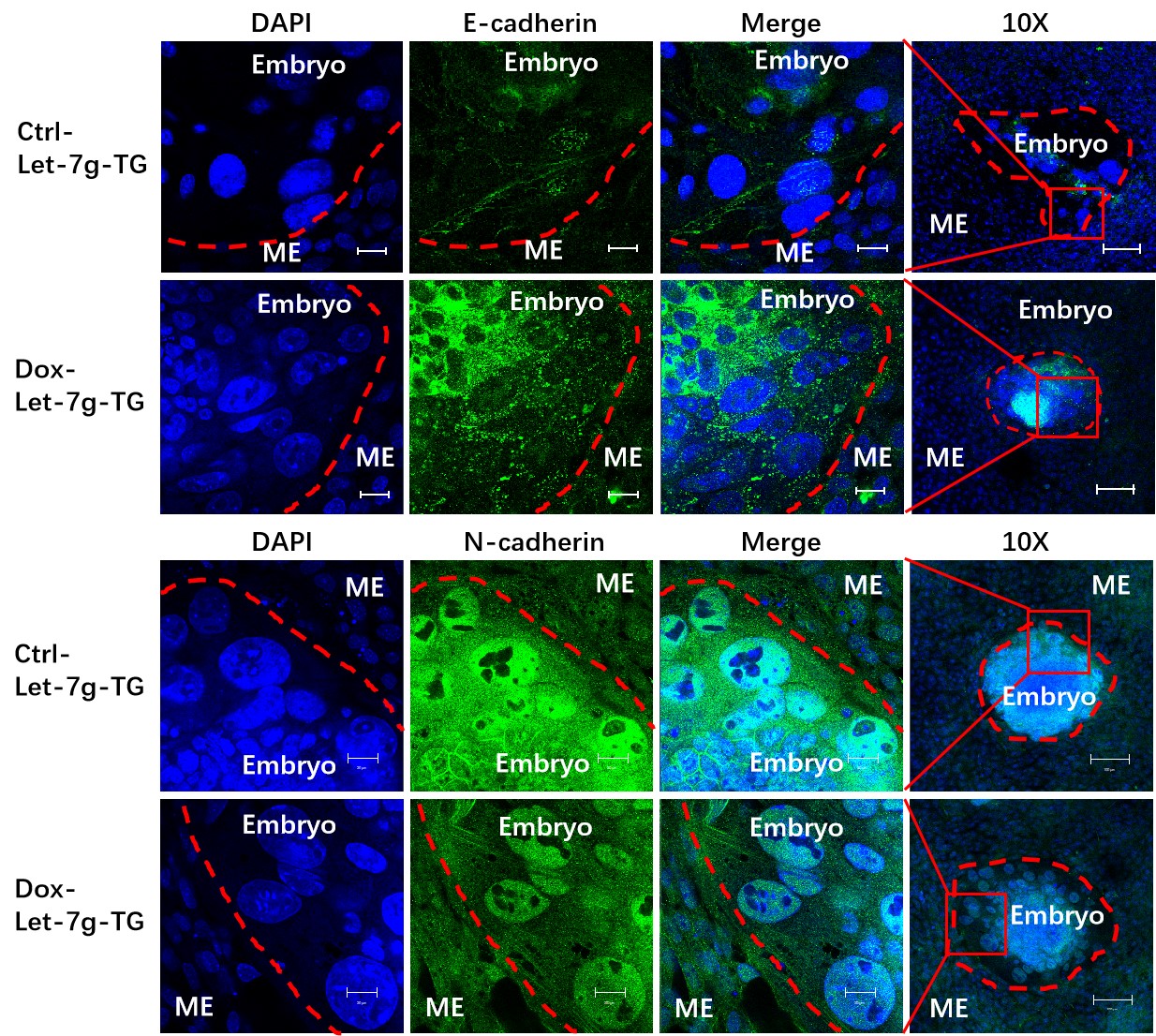

Supplement: Supplementary file 4 — Additional file 3. [file 12964_2020_562_MOESM4_ESM.jpg]
